# Reorganization of complex ciliary flows around regenerating *Stentor coeruleus*

**DOI:** 10.1098/rstb.2019.0167

**Published:** 2019-12-30

**Authors:** Kirsty Y. Wan, Sylvia K. Hürlimann, Aidan M. Fenix, Rebecca M. McGillivary, Tatyana Makushok, Evan Burns, Janet Y. Sheung, Wallace F. Marshall

**Affiliations:** 1 Living Systems Institute, University of Exeter, Stocker Road, Exeter EX4 4QD, UK; 2 Department of Molecular and Cellular Biology, Harvard University, Cambridge, MA 02138, USA; 3 Department of Biochemistry and Biophysics, UCSF, San Francisco, CA 94143, USA; 4 Department of Pathology, University of Washington, WA 98109, USA; 5 Center for Cardiovascular Biology, University of Washington, WA 98109, USA; 6 Department of Biology, Vassar College, NY 12604, USA; 7 Department of Physics and Astronomy, Vassar College, NY 12604, USA; 8 Marine Biological Laboratory, Physiology Course, Woods Hole, MA 02543, USA; 9 Marine Biological Laboratory, Whitman Center, Woods Hole, MA 02543, USA

**Keywords:** cilia coordination, metachronal waves, regeneration, morphogenesis, ciliary flows, *Stentor*

## Abstract

The phenomenon of ciliary coordination has garnered increasing attention in recent decades and multiple theories have been proposed to explain its occurrence in different biological systems. While hydrodynamic interactions are thought to dictate the large-scale coordinated activity of epithelial cilia for fluid transport, it is rather basal coupling that accounts for synchronous swimming gaits in model microeukaryotes such as *Chlamydomonas.* Unicellular ciliates present a fascinating yet understudied context in which coordination is found to persist in ciliary arrays positioned across millimetre scales on the same cell. Here, we focus on the ciliate *Stentor coeruleus*, chosen for its large size, complex ciliary organization, and capacity for cellular regeneration. These large protists exhibit ciliary differentiation between cortical rows of short body cilia used for swimming, and an anterior ring of longer, fused cilia called the membranellar band (MB). The oral cilia in the MB beat metachronously to produce strong feeding currents. Remarkably, upon injury, the MB can be shed and regenerated de novo. Here, we follow and track this developmental sequence in its entirety to elucidate the emergence of coordinated ciliary beating: from band formation, elongation, curling and final migration towards the cell anterior. We reveal a complex interplay between hydrodynamics and ciliary restructuring in *Stentor*, and highlight for the first time the importance of a ring-like topology for achieving long-range metachronism in ciliated structures.

This article is part of the Theo Murphy meeting issue ‘Unity and diversity of cilia in locomotion and transport’.

## 1. Introduction

‘When the anterior part is open, one may perceive about its Edges a very lively Motion; and when the Polyps presents itself in a certain manner, it discovers, on either side of these edges of its anterior part, somewhat very much resembling the wheels of a little Mill, that move with great velocity.’

        A. Trembley F.R.S. 1744 ([1], p.173.)

### (a) Long-range ciliary coordination in a unicellular organism

Cilia are ubiquitous appendages that occur in many different organisms. Cilia-mediated flows are important for microscale transport and propulsion, and have also been implicated in developmental patterning in early mammalian embryos [2]. The fundamental question of how cilia synchronize and interact is an open problem that has fascinated and confounded researchers for decades—its mechanistic specificity and physical origins have only become clearer in recent years (see [3] and references therein). Here, we propose and study a new model organism for ciliary coordination—the unicellular protist *Stentor*. Many protists present large-scale body coverings of cilia. Some even exhibit differentiation between short body cilia and larger compound or oral cilia. In *Stentor*, the oral cilia assume a prominent position at the anterior, forming a ring-like structure known as the *membranellar band* (MB), or peristome. Their collective beating stirs the fluid to generate strong feeding currents, producing an ‘alimentary vortex’ *sensu* Maupas [4], which draws food and other particulates towards the mouth of the organism. In a remarkable feat of cellular organization, the entire *Stentor* MB can be shed and regenerated anew [5].

We solicit *Stentor*'s capacity to regrow and replace lost structures to investigate how active, subcellular structures achieve long-range coordination over millimetric areas of surface. Few systems permit experimentally induced ciliogenesis and continuous, real-time monitoring of ciliary coordination as the cilia are regenerated *en masse*. The present study follows the growth and de novo development of the MB in *Stentor*. We combine state-of-the-art high-speed imaging and correlative flow field tracking to explore for the first time the extreme restructuring of cilia and extracellular flows during MB development. Arranged in dense, regularly stacked rows, oral cilia experience hydrodynamic coupling but are also connected by intracellular fibres. The dramatic structural and morphological changes accompanying MB regeneration give us a unique opportunity to assess how inter-ciliary cooperativity changes with length, connectivity, and even topology. We propose that the latter is a new mechanism associated with the attainment of global ciliary coordination in the oral apparatus of ciliates. Our results reveal a strong sensitivity of cilia coordination pattern to curvature, instantiating a natural cellular phenomenon in which boundaries play an essential role in ordering active non-equilibrium systems [6].

### (b) *Stentor*: the trumpet animalcule

**Figure 1.**
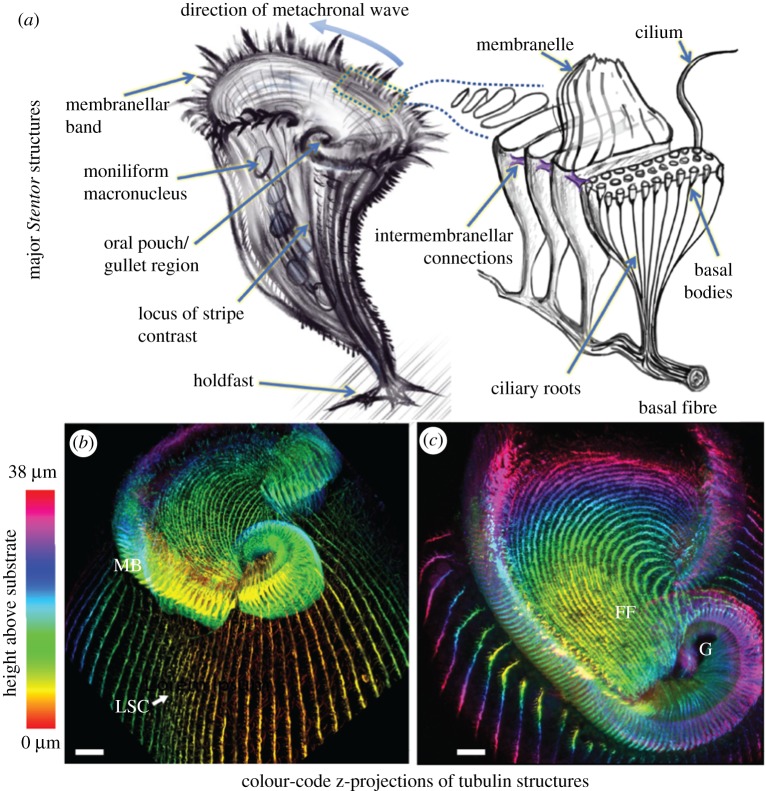
(*a*) Schematic of a single *Stentor* with key morphological features highlighted. The membranellar band [8] comprises rows of oral cilia arranged in parallel stacks (each approx. 7.5 µm × 1.5 µm). (*b*) Confocal immunofluorescence images highlighting the structure and organization of the membranellar band (MB), cortical striation patterns and associated rows of short body cilia. Note the abrupt change in width at the locus of stripe contrast (LSC). (*c*) Top view of the frontal field (FF) and gullet region (G). Antibody used in (*b,c*) was anti-α-tubulin (scalebars = 10 µm).

The first recorded account of *Stentor* was made by Abraham Trembley F.R.S, who in a letter to the president of the Royal Society [1, p.169], wrote ‘I have herewith the honour of transmitting to you the particulars of several observations I have made, during the course of the last summer, upon some species of very minute Water-Animals…’. (In fact, at the time Trembley had misidentified these organisms as *Hydra* [7].) *Stentor* are so-named for their trumpet-like morphology, in honour of a loud-voiced Greek hero who fought in the Trojan War. These unicellular ciliates occur widely in both freshwater and marine habitats, and exhibit both free-swimming and sedentary characteristics depending on environmental circumstance. Individual cells undergo dynamic and extensive shape changes owing to a highly contractile cortical structure, assuming a pear or tear-drop shape when free-swimming, contracting into a ball when disturbed, or else extending up to 1 mm in a rest state in which cells become attached to substrates via a posterior holdfast (figure 1*a*).

While diverse species of *Stentor* exist, we focus exclusively on *S. coeruleus Ehrenberg* 1830—a widely distributed species, noted for their striking cerulean pigmentation. *Stentor coeruleus*, also called the *Blue Stentor* [7,9], was favoured historically for cytological studies owing to its large size and moniliform macronucleus [10] and has been the subject of recent genomic studies [11]. The conical surface of the organism bears longitudinal stripes of distinctive colouration extending from the anterior all the way down to the tapered holdfast (figure 1*a*). The stripes have graded width around the cell; the region where stripe gradation becomes discontinuous is called the locus of stripe contrast (LSC), or ‘ramifying zone’. This region demarcates where the new oral primordium and associated field of basal bodies will form. The body cilia are inserted in rows along the clear stripes all over the body [10], and are subtended by contractile fibres called myonemes that mediate whole-body contractions. The MB encircles the broad anterior end of the organism, comprising an adoral zone of external cilia that are fused into rows of transverse plates termed membranelles (*sensu* [12]). In transmission electron microscopy (TEM) studies, Randall & Jackson [8] showed that these adoral structures were not individual large cilia but rather two or three rows of tightly packed cilia (20–25 cilia per row), so that each membranelle maintains functional unity as an intercalated ciliary sheath (figure 1*b*). One end of the MB terminates freely, while the other end spirals into a funnel-shaped invagination or gullet. Comprising around 250 stacked membranelles, the entire MB encloses the frontal field (FF), which is distinguished by a change in stripe orientation (figure 1*c*). The FF contains stripes that originated as ventral stripes that have migrated upwards over the course of regeneration (see §2).

### (c) Regenerative prowess of *Stentor*

A hallmark of life is its ability to repair, heal wounds, or replenish lost or damaged cellular structures [13,14]. Single cells are of special interest, for regeneration must necessarily take place entirely within the same cell. One classic example is flagellar regeneration in the biflagellate alga *Chlamydomonas*, where amputated flagella can be regrown to full length in about 1.5 h [15,16], whereupon biflagellar coordination also recovers [17,18]. But how do protists, which have to regenerate tens of thousands of cilia, recover from such injuries? *Stentor* was a favoured organism for studies of regeneration by early researchers owing to its large size, manoeuvrability, and remarkable regenerative capabilities [19]. Questioning the limits of cellular indivisibility led the likes of Lillie and Morgan [20,21] to conduct dissection experiments on *Stentor* to determine the minimal ingredients for regeneration. They found that the smallest fragments capable of regenerating into new, viable individuals were spheroids of only < 100 µm in diameter. Nucleated fragments, irrespective of whether they originate from the head or tail, are all capable of full regeneration. The oral apparatus (including MB, frontal field and mouth parts) is the most differentiated cortical landmark of *Stentor*, and is routinely regenerated. Two distinct physiological processes can elicit the same characteristic developmental sequence. In the first, a cell undertaking cell division will form a second oral primordium on its ventral surface, which eventually develops into the new oral apparatus for the posterior daughter cell. Meanwhile, the original oral structures are retained by the anterior daughter cell [22]. The second type of oral regeneration is induced by diverse treatments (such as dissections, grafting, chemical exposure), which lead to the removal or shedding of an existing oral apparatus and its gradual replacement by a new set of oral structures.

**Figure 2.**
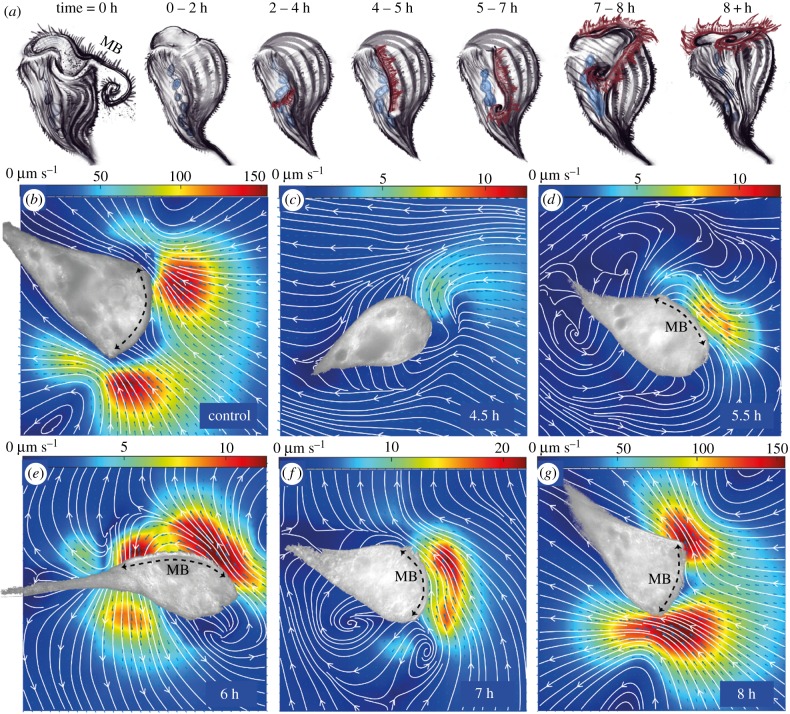
(*a*) Chronology of MB and flow restructuring during oral regeneration in *Stentor coeruleus* (see main text for details). At time 0, an existing MB was induced to shed by sucrose shock. After 1–2 h, a rift opens at the locus of stripe contrast. Between 2 and 5 h, oral cilia sprout, lengthen and eventually rearrange themselves into rows of stacked membranelles. After 5 h, the MB elongates and gradually migrates to assume a nearly circular structure at the anterior end. (*b–g*) particle image velocimetry (PIV) measurements of the extracellular flow fields associated with the regenerating MB for a control cell, and at the indicated times post sucrose shock for different regenerating individuals. (Colourmaps indicate flow speed, which changes significantly during regeneration. Brightfield images of the adhered organisms have been overlaid as masks on top of the flow maps. Black arrows label the MB location—wherever it is clearly identifiable.)

Here, we study only the second case: the injury-induced shedding and formation of a new MB. Initiation of regeneration requires the presence of at least one macronuclear node, some cytoplasmic material, as well as a piece of the microtubule cortex. Selective inhibition studies have shown that DNA-dependent RNA synthesis is required to supply the new protein needed during intermediate stages of regeneration, but not during the later stages. Depending on the temperature, medium and other unknown factors [23], the total regeneration time varies between 8 and 10 h at 20–25°C. Occasionally, regeneration is aborted prematurely and any partially formed structures are then resorbed [24]. A complete regeneration sequence proceeds through the following major stages (summarized in figure 2*a–g*). (i) Within the first hour, a rift forms at the site of the new primordium. (ii) Next, the oral cilia emerge, growing to approximately 0.5 µm, and new basal bodies form in the vicinity of existing body cilia. At this stage, the oral cilia are distributed randomly and are said to beat randomly. (iii) At 3–4 h, proliferation of basal bodies continues and the new primordium attains its definitive width of 12 µm. Meanwhile the oral cilia approach full length (though shorter ones are still evident) and some evidence of ciliary coordination is visible in scanning electron microscopy (SEM) images [25]. (iv) Oral cilia assemble into membranelles at 4–5 h. The new MB structure continues to lengthen and the macronucleus condenses. (v) At 6.5–7 h, the MB undergoes significant migration towards the anterior of the organism. (vi) Finally, after approximately 8 h, the newly formed MB curls all the way around the anterior end, and the nucleus also renodulates.

For this study, we induced regeneration in approximately 80 *Stentor* cells by sucrose shock, and can confirm all qualitative morphological features of the above staging. While diverse chemical treatments lead to a similar oral regeneration response [26], the sucrose shock was found to be the most reproducible—producing the cleanest MB removal, leaving sharp borders at the detachment zone. Here, we follow previously published protocols [11,27] with only slight modifications. Briefly, *Stentor* were exposed to a 10% sucrose solution for 2–3 min, which elicited synchronous MB shedding in more than 80% of the cases (electronic supplementary material). Cells were then pipetted into fresh medium to recover, and allowed to regenerate their MBs inside imaging chambers. We analysed the extracellular flows and ciliary activity accompanying regeneration on a cell-by-cell basis.

## 2. Reorganization of ciliary flows during membranellar band regeneration

In order to measure flow fields around *Stentor*, we developed a simple but effective protocol for preventing cell body motion, adapted from conventional cell attachment techniques. Glass-bottom Petri dishes used for imaging were pre-treated with Poly-D-Lysine to obtain substrate-adhered *Stentor* (electronic supplementary material). Contrary to many other methods trialled previously, ours had minimal effect on cell viability and did not adversely alter the oral regeneration dynamics. Our technique allowed continuous imaging at high spatio-temporal resolution over an extended period. The boundary conditions consisted of a free surface (a large open droplet) and a flat solid substrate. To obtain flow fields around the regenerating organisms, we seeded the medium with passive tracers (1 µm polystyrene beads) and performed particle image velocimetry (PIV) [28]. The body cilia (which do not undergo regeneration) remained motile but beat only intermittently throughout the oral regeneration process, and were capable of producing large-scale flows. We verified that the unsteady nature of body cilia activity also pertains to organisms that were not surface-adhered (electronic supplementary material, figure S1). This unsteadiness could underlie switching between swimming and feeding states: a stroke pattern optimized for feeding may reduce swimming efficiency [29], or *vice versa*. In marine trochophores, ciliary bands under nervous control can also reverse their beating direction depending on whether the organism is in a feeding or swimming state [30]. Here, in order to isolate flow contributions owing to oral cilia only, we restrict our discussion and analysis to recordings that do not show excessive body cilia motion or body contractions (electronic supplementary material, figure S2). Only flows that remained steady over 30 s recordings were used for the analysis of the flow fields.

### (a) Early regeneration and a linear membranellar band (0–5 h post sucrose shock)

The MB in the early stages of regeneration was particularly difficult to discern in the substrate-adhered organisms, as it is not possible to see the newly formed rift/MB until after a substantial structure has been formed. Sucrose-induced shedding rarely removes 100% of the membranelles—in most cases, a small number of these will remain deep inside the old gullet, destined to merge with the newly formed MB once the latter migrates successfully all the way to the anterior [7]. In these cases, the old membranelles can produce weak flows towards the old gullet (figure 2*c*). The new oral cilia reach a perceptible length at 4–5 h post sucrose shock, forming a linear band on the side of the organism.

### (b) Membranellar band growth and reorientation (5–7 h post sucrose shock)

As the oral cilia continue to grow, ciliary coordination increases concomitantly (see §3). The new MB is usually conspicuous under differential interference contrast (DIC) microscopy at 5 h after sucrose shock, producing localized flows that are directed posteriorly and largely parallel to the cell surface (figure 2*d*; electronic supplementary material, video S1). Over the next hour, the oral cilia proceed to lengthen and reorganize [25], producing flows that exhibit a greater perpendicular component that is directed away from the cell body (figure 2*e*). Flow magnitudes remain low during this period, on the order of 10 µm s^−1^. At about 7 h, the band structure migrates upwards and the new gullet begins to form, the cell anterior becomes more conical and distortion of streamlines is observed (figure 2*f*).

### (c) Emergence of the feeding vortex and completion of regeneration (more than 7 h post sucrose shock)

As the arc of the developing MB continues to move upward, its two ends eventually approach each other and the enclosed stripes bend into arcs in the FF (figures 1 and 2*a*). Finally, a characteristic double-vortex flow pattern emerges—as in the control organisms (compare figure 2*b,g*). In fully regenerated *Stentor*, the membranelles exhibit robust phase coordination and metachronal waves (MCW), the establishment of which is associated with a sharp transition to faster flows (on the order of 100 µm s^−1^) and with the appearance of the feeding vortex. Structurally similar flow fields were also measured in *Stentor* that have not been adhered to a substrate, but rather were attached spontaneously by their posterior holdfast (electronic supplementary material, figure S1). The morphology of these flow fields compares well with feeding flows around isolated *Vorticella* (a contractile, stalked ciliate that bears strong resemblance to *Stentor*) in the presence of a no-slip boundary [31,32].

## 3. Ciliary coordination and emergence of metachronal waves

### (a) Spatio-temporal correlations

**Figure 3.**
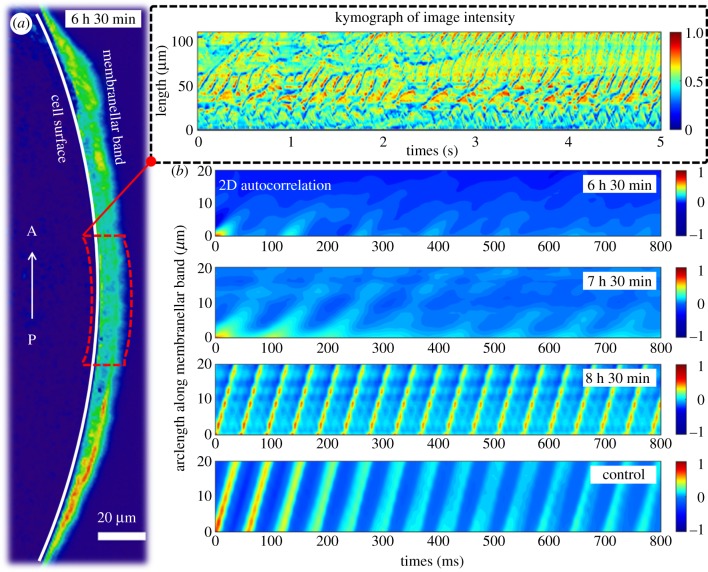
Ciliary coordination over the course of oral regeneration. (*a*) A motion heatmap was used to localize the beating cilia, e.g. to a narrow band on the surface of the organism (arrow in the direction of increasing arclength from posterior to anterior). A region of interest parallel to the ciliary band (red), was used to generate intensity kymographs. Inset: kymograph reveals local image structure and coherence. (*b*) The 2D intensity autocorrelation shows increasing ciliary coordination over the course of regeneration. Sustained MCWs (parallel lines of high correlation in P–A direction) only emerge once MB regeneration has been almost completed. Here, the slope of the parallel lines equals MCW speed. Labels indicate time post sucrose shock.

Next, we turned to high-speed imaging (frame rates of 1 kHz) to investigate how the spatial arrangement and beat synchronicity of the oral cilia contribute to the restructuring of extracellular flows during oral regeneration. Image sequences were processed to localize fast-moving ciliary structures (figure 3*a* and inset). Then, greyscale intensities measured from each region of interest were cross-correlated to determine how the spatiotemporal coordination exhibited by the beating membranelles changes over time.

At very early stages of regeneration, the short membranelles beat without proper coordination. Over the next 1–2 h, the growing ciliary band remains linear and incomplete waves of activity are propagated from the newly formed oral primordium towards the anterior (figure 3*b*, 6 h 30 min; electronic supplementary material, video S2). However, there is no long-range coordination at this stage, contrary to published observations [25]. Instead, characteristic wave-like structures appear and disappear intermittently along the MB, but rarely extending beyond one or two wavelengths (approx. 10 membranelles). As the reshaping structure begins to migrate towards the anterior, the MCWs become slightly more coordinated and sustained over much longer distances (figure 3*b*, 7 h 30 min). The membranelles in the developing MB propagate a diaplectic MCW (where the beat direction of individual membranelles is perpendicular to the wave direction) [33]. Hereafter, the ciliary beat amplitude no longer changes, but the beat frequency and speed of wave propagation remain subject to intracellular control [34]. Very late in regeneration, a single, highly coordinated MCW is propagated circularly from the gullet around the entire MB—and it is only then that global coordination of the membranelles is attained (electronic supplementary material, video S3). Existence of a single unidirectional wave is evident from plots of 2D intensity autocorrelations as a function of time and arclength along the developing MB. In the examples shown, both control *Stentor* and a very late-stage regenerating cell (8 h 30 min) show parallel lines of equal slope (figure 3*b*), corresponding to 0.58 mm s^−1^ and 0.57 mm s^−1^, respectively, for the speed of MCW propagation, and 16.1 Hz and 20.8 Hz respectively, for the mean beat frequencies of individual membranelles. The timing of the attainment of global ciliary coordination is also coincident with the emergence of the strong feeding vortex.

### (b) Infraciliature and hydrodynamic interactions

**Figure 4.**
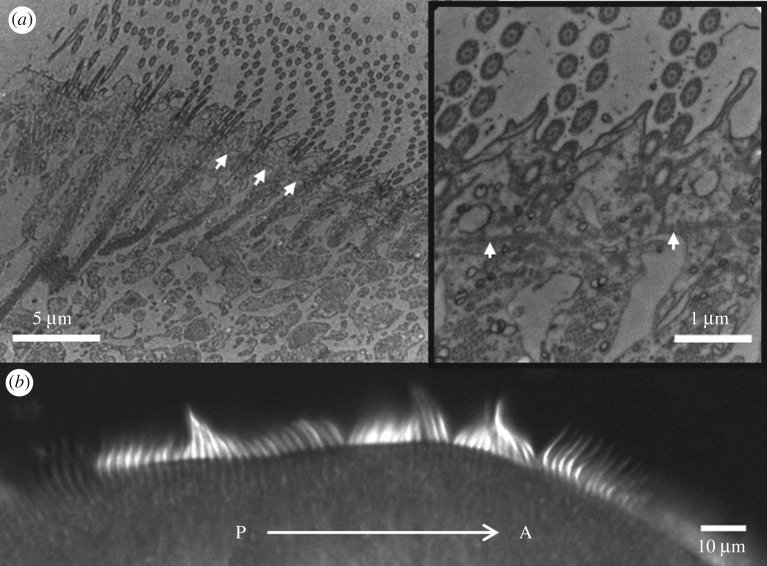
Correlating TEM sections with live-cell DIC microscopy at the same regeneration stage—at 6 h 45 min post sucrose-shock. (*a*) The ultrastructure is indistinguishable from control *Stentor,* fibrillar structures extend from the membranelles into the cytoplasm, in addition to transverse connections between neighbouring membranelles (arrows). (*b*) Cilia at the corresponding stage in live cells exhibit local but not global coordination—transient waves are propagated from the new oral primordium situated at the posterior (P), to the anterior end of the organism (A).

To further explain the lack of ciliary coordination early in regeneration and emergence of global order at late times, we compared our light microscopy data with TEM sections of fixed *Stentor* (figure 4; electronic supplementary material). Paulin & Bussey [25] showed previously that in the early stages of oral regeneration new oral cilia sprout from basal bodies that are arranged randomly in the anarchic field, consistent with the lack of ciliary coordination that we have observed. Between 4 and 5 h post sucrose shock these cilia undergo restructuring to assemble into regularly stacked rows of membranelles, with fibrillar structures connecting neighbouring membranelles also appearing at this stage (figure 1*a*, and fig. 32 from [8]). A rotation in direction of fluid pumping from longitudinal (with respect to the cell axis) to transverse (figure 2*d,e*) is fully consistent with the transition from a longitudinally directed beat pattern to a transversely directed beat [25]. We attribute the intermittent coordination between nearby membranelles observed at this stage (figure 3*b*) to hydrodynamic interactions: strong correlation at small spatial and temporal lags arising from strong coupling between nearby membranelles over distances of approximately 10 µm, in both intermediate (cilia have already reached full-length; electronic supplementary material, figure S3) and late-stage regenerating cells. This lengthscale is comparable to the bifurcation lengthscale for hydrodynamic coupling [35]. Further evidence of hydrodynamic effects can be deduced from Tartar's finding that evidence of ciliary coordination in grafted *Stentor* can be immediate—long before any new cellular connections could have formed between the new and old membranelles [7].

Though necessary, such passive fluid–structure interactions alone are insufficient to achieve global ciliary coordination. Instead, *Stentor* MCWs are regulated by excitatory signalling via a ‘silver line’ system of intermembranellar connections. It is suggested that the primordium-facing membranelles act as pacemakers [34]. As in some species of algal multiflagellates [36,37], calcium signalling via contractile elements can dynamically reverse the beating direction of the membranelles [38]. Addition of digitoxin and other chemicals directly modulates intermembranellar connectivity leading to a change in MCW wave velocity but affects the beat frequencies to a lesser extent [39]. The existence of a single MB frequency must require physical continuity across the entire MB. Indeed, in non-regenerating control *Stentor*, MCWs can be stopped or altered by severing or manipulating the basal fibres between membranelles [7,40]. These observations suggest that geometry is the final ingredient required to achieve global metachrony. By 7 h post sucrose shock, internal, fibrillar structures connecting the membranelles appear to be present (figure 4*a*) just as described in control *Stentor* [34]*,* yet coordination could not be sustained along the entire linear band of membranelles (figure 4*b*). An MCW with a unique, constant wave speed only emerges after the conical reshaping of the FF where the entire band becomes nearly circular.

## 4. Discussion and outlook

The formation of a new oral apparatus in the ciliate *Stentor* is a remarkable feat of organellar regeneration, differentiation and structural reorganization. It involves the proliferation of 15 000 individual basal bodies—their reconfiguration into a functional entity, along with a precise arrangement and migration of associated subpellicular microtubules, basal-body associated fibres and other contractile filaments. Here, we studied this process for the first time with correlative flow-tracking and quantitative high-speed imaging. Live-cell imaging is indispensable when inferring dynamics and behaviour, as erroneous or incomplete conclusions could be drawn from interpretation of static electron microscopy images alone. We developed a robust methodology to track how spatiotemporal coherence in the membranellar structure evolves in substrate-adhered organisms over the full course of oral regeneration (up to 10 h of continuous imaging). We showed the incoherent random beating of short oral cilia early in regeneration, followed by enhanced ciliary coordination and reorientation of fluid pumping, and ultimately the emergence of a single directional MCW spanning the MB that generates and sustains large-scale, vortical feeding flows.

As an emerging model organism with great potential for biophysical studies of ciliary regeneration and coordination, *Stentor* offers many novel avenues for consideration by the interested reader. The unique synchronization dynamics of *Stentor* membranelles add a new dimension to a growing literature on biophysical studies of interacting arrays of cilia in well-established model systems [37,41,42]. The present study shows that a precise geometric orientation of stacked membranelles is required to achieve global metachronal coordination, highlighting the importance of topology [43], curvature [44] and periodic boundary conditions [45] for the stabilization of MCWs on ciliated surfaces. This is *in addition* to attaining correct basal body and membranellar alignment, fibrillar connections and hydrodynamic interactions between full-length cilia. We suggest that for this reason *Stentor* oral regeneration always proceeds in the same manner in order to attain a highly specific, ultimate ciliary configuration that can effectively exploit interciliary hydrodynamic interactions [46–48] to enhance feeding flows. Indeed, incorrectly placed MBs are often resorbed [24]. Such looping ciliary arrays are also prevalent as ciliary bands in planktonic marine larvae [30,49].

Intracellular organization may also be important. Evidence of physical connections between the membranelles and other subcellular structures (figure 4) already implicates control of MCW activity by biochemical signalling. In models of circular spring networks, it has been shown that synchronization states depend on the topology of the coupling [50]. Detailed studies of *Stentor* infraciliature by serial EM reconstruction (beyond the chemical fixation techniques we have used here) will be required to elucidate the 3D connectivity between cilia, and between membranelles. This could be complemented by dissection studies to take advantage of the multifarious regenerative capabilities of *Stentor*. Targeted physical microsurgical manipulations including dissection, grafting or induced resorption can be used in conjunction to study ciliary and flow reorganization. It has been shown that oral regeneration can even be induced by cutting the intermembranellar fibres [51]. Further investigation of the specialized geometric constraints exhibited by *Stentor* membranellar cilia and their relation to the internal structure and dynamics of the MCWs will appear in a follow-up study.

The novelty of this system also presented a number of challenges. First, we were unable to obtain cell-cycle synchronized cultures. This, coupled with the highly contractile cell body, meant that experimental organisms were highly variable in size, shape and behaviour. Second, even though the dynamics concerned are intrinsically three-dimensional, for constraints of phototoxicity and imaging speed we used 2D-imaging methods only. Third, as part of this study, we had also intended to investigate an alternative form of oral regeneration termed *in situ* regeneration, in which oral cilia reputedly could be induced to sever at the transition zone and regrown in place (as in the biflagellate alga *Chlamydomonas* [52]). In this case, there is no morphological restructuring of the cytoskeleton [53,54]. *In situ* regeneration would have enabled the effective decoupling of intracellular or ultrastructural changes from passive hydrodynamic interactions during cilia and membranellar regrowth. After multiple attempts, the present authors were unable to reproduce this configuration—the oral cilia were always shed together with the entire MB.

Despite these sources of variability, our results demonstrate a remarkable consistency in the timing and dynamics of *Stentor* oral regeneration. Electing to use the sucrose shock method, we were able to induce reversible and simultaneous shedding of MBs in large sample sizes. Physiological regeneration, for instance, in response to injury of feeding organelles or excess mechanical stresses, also elicits a similar programmed response [10,55]. Thus, membranellar regeneration appears to be a universal mechanism by which unicellular protists repair and refresh their oral structures [56]. The concomitant change in topology from a linear to a circular MB appears to be critical for attainment of global coordination of membranelles and the inward-directed feeding flows. In this regard, how protists such as *Stentor* achieve great complexity of cortical organization and internal control over locomotor appendages, could hold the key to understanding the evolution of neural circuitry that is specialized for maintaining coherence of ciliary arrays [49,57].

## Supplementary Material

Supplementary information and figures

## Supplementary Material

Ciliary flows during regeneration

## Supplementary Material

Ciliary dynamics at 6.5 hrs

## Supplementary Material

Ciliary dynamics at 8.5 hrs

## Data accessibility

For sample videos and detailed methodology, please refer to the electronic supplementary material uploaded as part of this article. Code used for the analysis can be found at https://github.com/shurlimann/stentor-cilia-autocorrelation). Additional datasets are available at https://doi.org/10.5281/zenodo.3590430.

## References

[1] Trembley A 1744 Translation of a letter from Mr Abraham Tembley, FRS, to the President, with observations upon several newly discovered species of fresh water polyps. Phil. Trans. R. Soc. 43, 169–183. (10.1098/rstl.1744.0040)

[2] Smith DJ, Montenegro-Johnson TD, Lopes SS 2019 Symmetry-breaking cilia-driven flow in embryogenesis. Annu. Rev. Fluid Mech. 51, 105–128. (10.1146/annurev-fluid-010518-040231)

[3] Wan KY 2018 Coordination of eukaryotic cilia and flagella. Essays Biochem. 62, 829–838. (10.1042/EBC20180029) 30464007 PMC6281475

[4] Maupas E 1888 Recherches experimentales sur la multiplication des infusoires cilies. Archives de Zoologie Experimentale et Generale 46, 498–516.

[5] Moxon W 1869 On some points in the anatomy of *Stentor* and its mode of division. J. Anat. Physiol. 3, 279–293. PMC1318683 17230806

[6] Wu K-T, Hishamunda JB, Chen DTN, DeCamp SJ, Chang Y-W, Fernández-Nieves A, Fraden S, Dogic Z 2017 Transition from turbulent to coherent flows in confined three-dimensional active fluids. Science 355, eaal1979 (10.1126/science.aal1979) 28336609

[7] Tartar V 1961 The biology of Stentor. Oxford, UK: Pergamon Press LTD (10.1016/C2013-0-01654-4)

[8] Randall JT, Jackson SF 1958 Fine structure and function in *Stentor polymorphus*. J. Cell Biol. 4, 807–830. (10.1083/jcb.4.6.807) PMC2224530 13610947

[9] Slabodnick MM, Marshall WF 2014 Stentor coeruleus. Curr. Biol. 24, R783–R784. (10.1016/j.cub.2014.06.044) 25202864 PMC5036449

[10] Johnson HP 1893 A contribution to the morphology and biology of the *Stentors*. J. Morphol. 8, 467–562. (10.1002/jmor.1050080303)

[11] Sood P, McGillivary R, Marshall WF 2017 The Transcriptional Program of Regeneration in the Giant Single Cell, *Stentor coeruleus*. *bioRxiv* (10.1101/240788)

[12] Sterki V 1878 Beiträge zur Morphologie der Oxytrichinen. Zeitschrift fur wissenschaftliche Zoologie 31, 29–58. (https://www.zobodat.at/pdf/Zeitschrift-fuer-wiss-Zoologie_31_0029-0058.pdf)

[13] Tang SKY, Marshall WF 2017 Self-repairing cells: how single cells heal membrane ruptures and restore lost structures. Science 356, 1022–1025. (10.1126/science.aam6496) 28596334 PMC5664224

[14] Reddien PW, Alvarado AS 2004 Fundamentals of Planarian regeneration. Annu. Rev. Cell Dev. Biol. 20, 725–757. (10.1146/annurev.cellbio.20.010403.095114) 15473858

[15] Rosenbaum JL, Moulder JE, Ringo DL 1969 Flagellar elongation and shortening in *chlamydomonas*: the use of Cycloheximide and Colchicine to study the synthesis and assembly of flagellar proteins. J. Cell Biol. 41, 600–619. (10.1083/jcb.41.2.600) 5783876 PMC2107765

[16] Hendel NL, Thomson M, Marshall WF 2018 Diffusion as a ruler: modeling kinesin diffusion as a length sensor for intraflagellar transport. Biophys. J. 114, 663–674. (10.1016/j.bpj.2017.11.3784) 29414712 PMC5985012

[17] Wan KY, Leptos KC, Goldstein RE 2014 Lag, lock, sync, slip: the many ‘phases’ of coupled flagella. J. R. Soc. Interface. 11, 20131160 (10.1098/rsif.2013.1160) 24573332 PMC3973360

[18] Goldstein RE, Polin M, Tuval I 2011 Emergence of synchronized beating during the regrowth of eukaryotic flagella. Phys. Rev. Lett. 107, 148103 (10.1103/PhysRevLett.107.148103) 22107238

[19] De Terra N 1966 Leucine incorporation into the membranellar bands of regenerating and nonregenerating *Stentor*. Science 153, 543–545. (10.1126/science.153.3735.543) 17830372

[20] Lillie FR 1896 On the smallest parts of *Stentor* capable of regeneration; a contribution on the limits of divisibility of living matter. J. Morphol. 12, 239–249. (10.1002/jmor.1050120105)

[21] Morgan TH 1901 Regeneration of proportionate structures in *Stentor*. Biol. Bull. 2, 311–328. (10.2307/1535709)

[22] James EA 1967 Regeneration and division and in *Stentor coeruleus*: the effects of microinjected and externally applied actinomycin D and puromycin. Dev. Biol. 16, 577–593. (10.1016/0012-1606(67)90065-6) 4967265

[23] Burchill BB 1968 Synthesis of RNA and protein in relation to oral regeneration in the ciliate *Stentor coeruleus*. J. Exp. Zool. 167, 427–438. (10.1002/jez.1401670405)

[24] Tartar V 2019 Induced resorption of oral primordia in regenerating *Stentor coeruleus*. J. Exp. Zool. 139, 1–31. (10.1002/jez.1401390103) 13654665

[25] Paulin JJ, Bussey J 1971 Oral regeneration in the ciliate *Stentor coeruleus*: a scanning and transmission and electron optical study. J. Protozool. 18, 201–213. (10.1111/j.1550-7408.1971.tb03308.x) 4997035

[26] Tartar V 1957 Reactions of *Stentor coeruleus* to certain substances added to the medium. Exp. Cell Res. 13, 317–332. (10.1016/0014-4827(57)90011-3) 13480299

[27] Lin A, Makushok T, Diaz U, Marshall WF 2018 Methods for the study of regeneration in *Stentor*. J. Visualized Exp. 136, e57759 (10.3791/57759) PMC6101732 29985325

[28] Thielicke W, Stamhuis EJ 2014 PIVlab—towards user-friendly, affordable and accurate digital particle image velocimetry in MATLAB. J. Open Res. Softw. 2, e30 (10.5334/jors.bl)

[29] Strathmann RR, Grunbaum D 2006 Good eaters, poor swimmers: compromises in larval form. Integr. Comp. Biol. 46, 312–322. (10.1093/icb/icj031) 21672744

[30] Marinkovic M, Berger J, Jékely G 2019 Neuronal coordination of motile cilia in locomotion and feeding. Phil. Trans. R. Soc. B 375, 20190165 (10.1098/rstb.2019.0165) 31884921 PMC7017327

[31] Sleigh MA, Barlow DI 1976 Collection of food by *Vorticella*. Trans. Am. Microsc. Soc. 95, 482-486. (10.2307/3225140)

[32] Pepper RE, Roper M, Ryu S, Matsudaira P, Stone HA 2010 Nearby boundaries create eddies near microscopic filter feeders. J. R. Soc. Interface 7, 851–862. (10.1098/rsif.2009.0419) 19942677 PMC2874229

[33] Knight-Jones EW 1954 Relations between metachronism and the direction of Ciliary Beat in Metazoa. J. Cell Sci. s3-95, 503–521. (https://jcs.biologists.org/content/s3-95/32/503)

[34] Sleigh MA 1957 Further observations on co-ordination and the determination of frequency in the Peristomial Cilia of *Stentor*. J. Exp. Biol. 34, 106–115.

[35] Brumley DR, Wan KY, Polin M, Goldstein RE 2014 Flagellar synchronization through direct hydrodynamic interactions. Elife 3, e02750 (10.7554/eLife.02750) 25073925 PMC4113993

[36] Wan KY, Goldstein RE 2018 Time irreversibility and criticality in the motility of a flagellate microorganism. Phys. Rev. Lett. 121, 058103 (10.1103/PhysRevLett.121.058103) 30118294 PMC7616082

[37] Wan KY, Goldstein RE 2016 Coordinated beating of algal flagella is mediated by basal coupling. Proc. Natl Acad. Sci. USA 113, E2784–E2793. (10.1073/pnas.1518527113) 27140605 PMC4878519

[38] Bannister LH, Tatchell EC 1968 Contractility and the fibre systems of *Stentor coeruleus*. J. Cell Sci. 3, 295–308. 4969426 10.1242/jcs.3.2.295

[39] Sleigh MA 1956 Metachronism and frequency of beat in the peristomial cilia of *Stentor*. J. Exp. Biol. 33, 15–28.

[40] Worley LG 6 2019 Ciliary metachronism and reversal in paramecium, spirostomum and *Stentor*. J. Cell Comp. Physiol. 5, 53–72. (10.1002/jcp.1030050105)

[41] Gheber L, Priel Z 1989 Synchronization between beating cilia. Biophys. J. 55, 183–191. (10.1016/S0006-3495(89)82790-0) 2930819 PMC1330453

[42] Feriani L, Juenet M, Fowler CJ, Bruot N, Chioccioli M, Holland SM, Bryant CE, Cicuta P 2017 Assessing the collective dynamics of motile cilia in cultures of human airway cells by multiscale DDM. Biophys. J. 113, 109–119. (10.1016/j.bpj.2017.05.028) 28700909 PMC5510766

[43] Nawroth JC, Guo H, Koch E, Heath-Heckman EAC, Hermanson JC, Ruby EG, Dabiri JO, Kanso E, McFall-Ngai M 2017 Motile cilia create fluid-mechanical microhabitats for the active recruitment of the host microbiome. Proc. Natl Acad. Sci. USA 114, 9510–9516. (10.1073/pnas.1706926114) 28835539 PMC5594677

[44] Nasouri B, Elfring GJ 2016 Hydrodynamic interactions of cilia on a spherical body. Phys. Revi. E 93, 033111 (10.1103/PhysRevE.93.033111) 27078451

[45] Niedermayer T, Eckhardt B, Lenz P 2008 Synchronization, phase locking, and metachronal wave formation in ciliary chains. Chaos 18, 037128 (10.1063/1.2956984) 19045502

[46] Han J, Peskin CS 2018 Spontaneous oscillation and fluid–structure interaction of cilia. Proc. Natl Acad. Sci. USA 115, 4417–4422. (10.1073/pnas.1712042115) 29632178 PMC5924875

[47] Elgeti J, Gompper G 2013 Emergence of metachronal waves in cilia arrays. Proc. Natl Acad. Sci. USA 110, 4470–4475. (10.1073/pnas.1218869110) 23487771 PMC3607033

[48] Brumley DR, Polin M, Pedley TJ, Goldstein RE 2015 Metachronal waves in the flagellar beating of volvox and their hydrodynamic origin. J. R. Soc. Interface 12, 20141358 (10.1098/rsif.2014.1358) 26040592 PMC4528573

[49] Gilpin W, Prakash VN, Prakash M 2016 Vortex arrays and ciliary tangles underlie the feeding–swimming trade-off in starfish larvae. Nat. Phys. 13, 380–386. (10.1038/nphys3981)

[50] Heidemann KM, Sageman-Furnas AO, Sharma A, Rehfeldt F, Schmidt CF, Wardetzky M 2018 Topology counts: force distributions in circular spring networks. Phys. Rev. Lett. 120, 068001 (10.1103/PhysRevLett.120.068001) 29481239

[51] De Terra N 1985 Does the oral apparatus of the ciliate *Stentor* inhibit oral development by release of a diffusible substance? J. Embryol. Exp. Morphol. 87, 241–247. 3928798

[52] Marshall W, Qin H, Rodrigo Brenni M, Rosenbaum J 2005 Flagellar length control system: testing a simple model based on intraflagellar transport and turnover. Mol. Biol. Cell 16, 270–278. (10.1091/mbc.e04-07-0586) 15496456 PMC539171

[53] Shigenaka Y, Yamaoka T, Ito Y, Kaneda M 1979 An electron and microscopical study and on ciliary detachment and reformation in a heterotrichous ciliate, *Stentor ceoeruleus*. J. Electron Microsc. 28, 73–82. (10.1093/oxfordjournals.jmicro.a050165)

[54] Tartar V 1968 Regeneration in situ of membranellar cilia in *Stentor coeruleus*. Trans. Am. Microsc. Soc. 87, 297–306. (10.2307/3224813)

[55] Stevens NM 1903 Notes on regeneration in *Stentor coeruleus*. Archiv für Entwicklungsmechanik der Organismen 16, 461–475. (10.1007/BF02152028)

[56] Bakowska J, Marlo Nerlon E, Frankel J 1982 Development of the ciliary and pattern of the oral and apparatus and of *Tetrahymena thermophila*. J. Protozool. 29, 366–382. (10.1111/j.1550-7408.1982.tb05416.x)

[57] Veraszto C, Ueda N, Bezares-Calderon LA, Panzera A, Williams EA, Shahidi R, Jekely G 2017 Ciliomotor circuitry underlying whole-body coordination of ciliary activity in the *Platynereis* larva. eLife 6, e26000 (10.7554/eLife.26000) 28508746 PMC5531833

