# Supplementary material for: Reorganization of complex ciliary flows around regenerating Stentor coeruleus
Source: Philos Trans R Soc Lond B Biol Sci. 2019 Dec 30;375(1792):20190167. doi: 10.1098/rstb.2019.0167 (PMC7017328; doi:10.1098/rstb.2019.0167)
Supplement: Supplementary information and figures [file rstb20190167supp1.pdf]

Supplementary Material

In the following, we provide further details of the experimental and analysis protocols.

1. Cell culture.
Wild *Stentor coeruleus* cells were isolated from a pond at Golden Gate Park in San Francisco, California, USA (37°46'13.0"N 122°30'08.5"W) on 12 January 2018. Individual cells were manually isolated from the pond water and cultured as in [25].

2. Immunofluorescence.
Methanol Fixation: About 10 healthy *Stentor* cells were collected in a 1.5 mL centrifuge tube and centrifuged at 800 rpm for 3 min. Cells were then re-suspended in -80 °C methanol, incubated for 30 min at -80 °C, washed 3 times with room temperature PBS, and blocked with 2% BSA in PBS for 1 h at room temperature. The primary antibody, monoclonal anti-α-tubulin antibody (mouse) (Fisher Scientific, # T5168-100UL), was incubated for 1 hr at room temperature in 2% BSA in PBS, followed by 3 washes in PBS. The secondary antibody, goat anti-mouse, Alexa Fluor Plus 488 (Fisher Scientific, # A32723), diluted in 2% BSA in PBS, was incubated for 1 hr at room temperature. Finally, cells were washed 3 times with PBS and mounted between two #1.5 glass coverslips with a small volume (~40 µL) of mounting media (VECTASHIELD, Vector Laboratories). The coverslips were separated with a spacer made using the CultureWell silicone sheet material (Grace Bio Labs, 664475), with the sample chamber made using a 3/16'' hole punch.

Light Microscopy: Cells were imaged using Zeiss 880 laser scanning confocal microscope with Airyscan at 60x magnification, with 2 µm spacing between z-slices. For color-coded 3D rendering in Figure 1b and c, the spectrum LUT for temporal-color coded hyperstacks (Temporal-Color Code plugin) was used from FIJI (Fiji is Just ImageJ).

3. Cell substrate adherence.
Poly-D-lysine (Millipore, A-003-E) was diluted to a final concentration of 0.02 mg/mL in pasteurized springwater (Carolina Biological Supply Company, # 132458). Glass bottom dishes (MatTek Corporation, P35G-1.5-14-C, 35 mm in diameter) were coated and incubated with the poly-D-lysine solution for at least 2 hrs at 37 °C. After incubation, the dishes were washed 3 times with pasteurized springwater to remove excess poly-D-lysine. Care was taken to ensure that the dishes did not fully dry out. Cells were collected via pipette aspiration and gently placed on the coated glass bottom dishes. After a few minutes, the cells settled and remained stationary on the bottom of the dish.

4. Shedding of membranellar band.
For each experiment, between 5 and 20 *Stentor* cells were isolated from the culture and gently washed using pasteurized springwater (Carolina Biological Supply Company, # 132458). The organisms were then transferred to a solution of sucrose in pasteurized spring water (to a final concentration of 10% sucrose). After 2 min, the majority of cells were confirmed under a low-power dissecting scope to have shed their membranellar bands. These cells were then gently washed 3 times with fresh pasteurized springwater to remove the sucrose. *Stentor* were observed to undergo normal regeneration after loss of the MB in the presence of both the poly-D-lysine coated coverslips and 1 µm polystyrene beads (see Section 5), compared to control organisms that were allowed to undergo regeneration in pasteurized springwater without beads in a dish that was not coated with poly-D-lysine.

5. Protocol for light microscopy of live cells.
DIC imaging and flow field tracking: For flow field studies, we seeded the medium (pasteurized springwater, Carolina Biological Supply Company, # 132458) with 1 µm polystyrene microspheres (2% solids, ThermoFisher) which were pre-washed in springwater, to a final dilution of ~1/500. Regenerating organisms were pipetted gently onto the pre-coated MatTek dishes, and allowed to settle before imaging. Each dish contained up to 4 cells, in an open droplet of ~1 ml in volume. Care was taken to ensure cells were spaced far enough apart that the flows generated by each individual could be considered independent of the others in the same dish.

Imaging was performed with a 10x objective with DIC (for enhanced contrast of ciliary structures and beads) on a Nikon inverted microscope (TiE) with humidity control to minimise evaporation. Fast time-lapse imaging (50 or 100 frames per second, recordings of 30 s duration) was performed at regular intervals (approximately hourly) during the regeneration process, using a Hamamatsu ORCA-Flash4.0 V3 camera. To analyse flow, cell body contours were first segmented and masked, and then flow fields were tracked using standard Particle

Image Velocimetry (PIV) methods with the Open Source software PIVlab [26]. Flow fields were post-processed to determine average flow parameters and streamlines using custom MATLAB code (the function streamslice was used to draw and display streamlines). For surface-adhered organisms, 2D flow fields were consistently imaged at a height approximately midway between the substrate and surface of the organism (usually 100-150 µm above the substrate).

High-speed imaging and MCW analysis: Substrate-adhered organisms used for high-speed imaging of the cilia were imaged under conventional light microscopy (Zeiss Axiovert). Videos were obtained at 1000 frames per second with a high-speed camera (Phantom Miro ex4, Vision Research) with a 20x objective, with or without an additional 1.6x tube lens, and post-processed with custom code written in Python and Matlab. Sample code has been uploaded to GitHub and is available here https://github.com/shurlimann/stentor-cilia-autocorrelation. Briefly, a motion heatmap based on pixel intensity standard deviation was used to manually define a 1D coordinate system along the beating membranellar band that allowed us to compute 2D kymographs of image intensity.

6. Transmission Electronmicroscopy of fixed specimens.
Organisms were fixed for 1 h with a cold mixture of 2 parts of 0.05M cacodylate buffer (pH 7.4), 1 part of 4% osmic acid, and 1 part of 6% glutaraldehyde on ice. The cells were then washed once for 20 min with cold 0.05M cacodylate buffer (pH 7.4) and kept on ice overnight. For electronmicroscopy imaging, cells were embedded in agar blocks, dehydrated in an ethanol series, and embedded in Durcupan (Sigma), as described previously in D. Włoga *et al*, Eukaryotic Cell, 2008 7(8); (DOI: 10.1128/EC.00085-08). Ultrathin sections were contrasted with Reynolds lead citrate and uranyl acetate.

7. Extracellular flow fields around non-adherent *Stentor*.
Additional experiments were performed using a different *S. coeruleus* strain (Carolina Biological Supply Company, #131598). Organisms were imaged in pasteurized springwater (Carolina Biological Supply Company, # 132458), at 30 fps over 30 frames. Sample chambers used were much larger compared to the size of the organisms (3/16'' diameter holes punched in Grace Biolabs secure seal adhesive sheet (sku 620001). Flow fields in this case were also processed using PIVlab. As expected, the proximity of the solid boundary did affect the fluid drag, so that adhered organisms produced slower flows in comparison to Figure S1. However, these additional results show that in *Stentor* that are attached naturally by its posterior holdfast and that had intact membranellar bands, surface-adherence did not change the qualitative character of the ciliary flows.

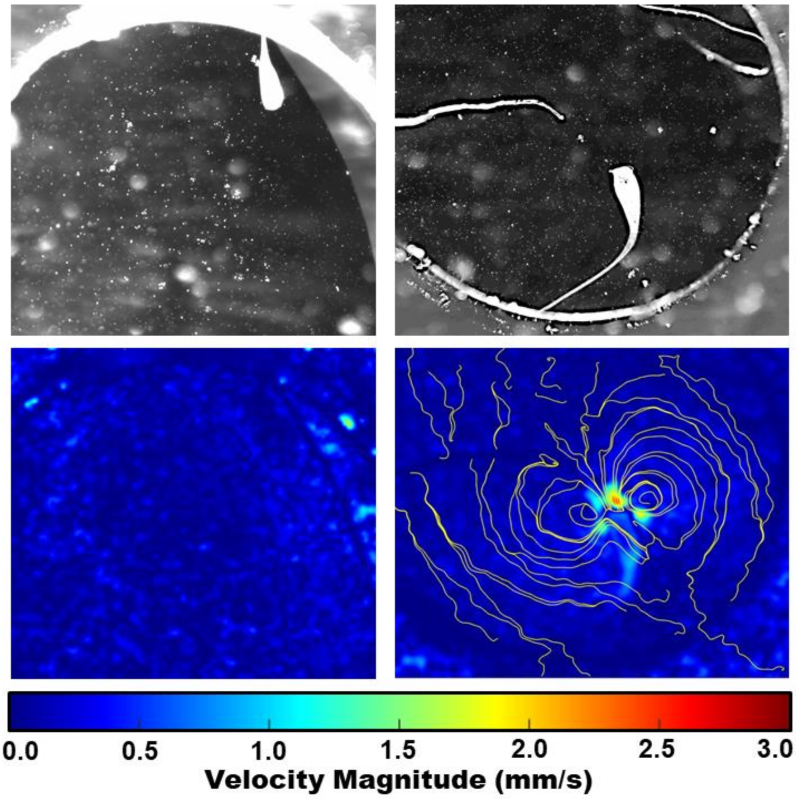

Figure S1: Extracellular flow fields around *Stentor* attached naturally by its posterior holdfast but with the body free to move (not substrate-adhered). Left column: A semi-contracted cell observed when the cilia are at rest, and no fluid motion is apparent. Right column: the same cell observed in a fully extended state at a later time, where vigorously beating membranellar cilia produce a characteristic double-vortex pattern of streamlines.

8. Excess body cilia motion and body contractions.
As discussed in the main text, *Stentor* exhibit highly variable behavioural dynamics even during free-swimming (see also Figure S1 above), including dramatic contractions of the body. This is often accompanied by fast beating of the body cilia and flow reversals. In order to isolate flows due to the oral cilia only, we considered only steady-state behaviour. Given the high variability in the shape and orientation of the organisms, we did not perform averaging of near-field flows around different cells at the same time point but focused on the average flow pattern for each individual at a given time point. Data in which cells displayed erratic behaviour was excluded from our analysis of the regeneration dynamics. Two specific examples of excluded data are given below.

a) Figure S2 shows a cell that was imaged 2 hours 40 minutes after complete MB shedding via sucrose shock. There are no oral cilia present at this stage. We see that the body cilia alone are sufficient to generate highly dynamic flows which change unpredictably over time.

b) Figure S3 shows a cell that was imaged at 6 hrs 30 min and 7 hrs 30 min post sucrose-shock. The regenerating MB is already visible at both time points and already present in an anterior position. Again, body distortions and body cilia activity produced dynamic flows, masking the contribution of the oral cilia.

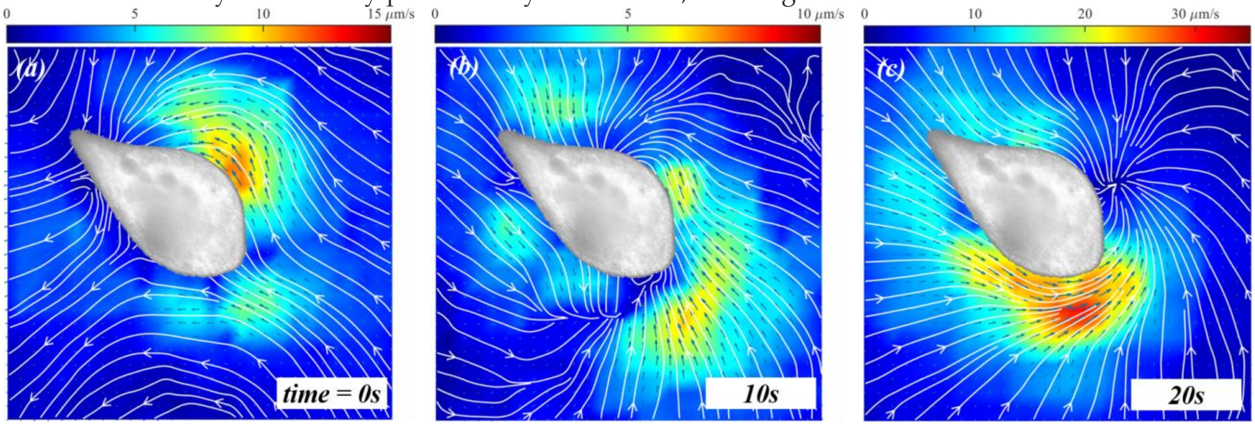

Figure S2: In the absence of the MB/oral cilia (2 hrs 40 min post sucrose shock), the body cilia can produce highly dynamic flows. Shown are snapshots of the flow field pattern (averaged over 2s) at three different times separated by 10s.

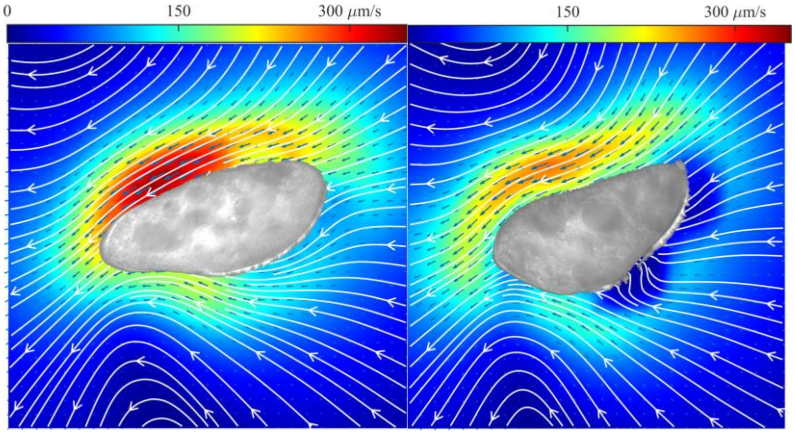

Figure S3: Excess body cilia motion and body contractions can distort and overwhelm the flows generated by the oral cilia in the developing membranellar band. Flow fields shown were obtained at 6.5 hrs (left) and 7.5 hrs (right) post sucrose shock, respectively.

9. Growth kinetics of membranellar cilia.
Oral cilia lengths were measured over the course of regeneration. As described in the text, the location of the new oral primordium is not visible until a band structure has already formed -- so length measurements start from a minimum of ~5 µm. The band grows heterogeneously: the cilia located in the centre grow faster than those on the sides of the band (see also the EM data from [23]). Here, length measurements were done by hand in FIJI at 5 separate locations along the membranellar band and averaged. Due to the high degree of variability in morphology it was not possible to compute these lengths automatically without manual input.

To determine approximate timescales for ciliary regrowth, we plotted the measured oral cilia lengths as a function of time, and overlaid as a rough guide (no fitting) with the form determined by the balance-point model for flagellar regrowth [16,49]. This model predicts the following functional dependence:

$$t = t_0 - \tau(L/L_0 + \ln(1 - L/L0))$$

Here, t_0 = 260 min (delay time before the oral cilia growth begins), τ = 450 min (total regeneration time), L_0= 30 μm (final length of oral cilia).

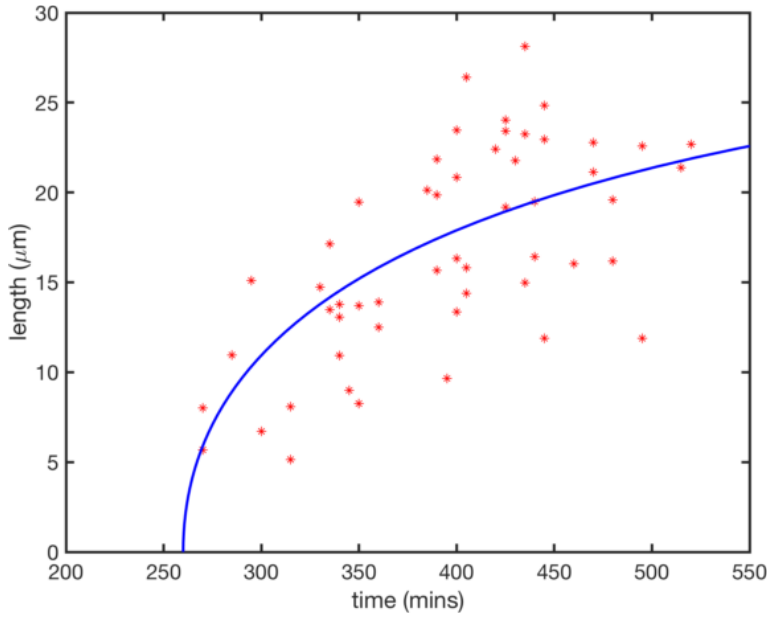

Figure S4: Regrowth kinetics of the *Stentor* membranellar band. The length of oral cilia is plotted against the time elapsed since membranellar band shedding via sucrose shock.

10. Additional flow field examples.

Figure 2 (main text) shows representative flow fields measured at different timepoints over the course of regeneration. Here we present some additional examples. In particular, Figure S5 g-i correspond to completed regeneration.

11. Availability of raw data

We include full video files of the PIV experiments (Vimeo link tbc) corresponding to Figures 2 b-g, which represent the extracellular flows generated by MB in cells at different stages of MB regeneration, and the control organism.

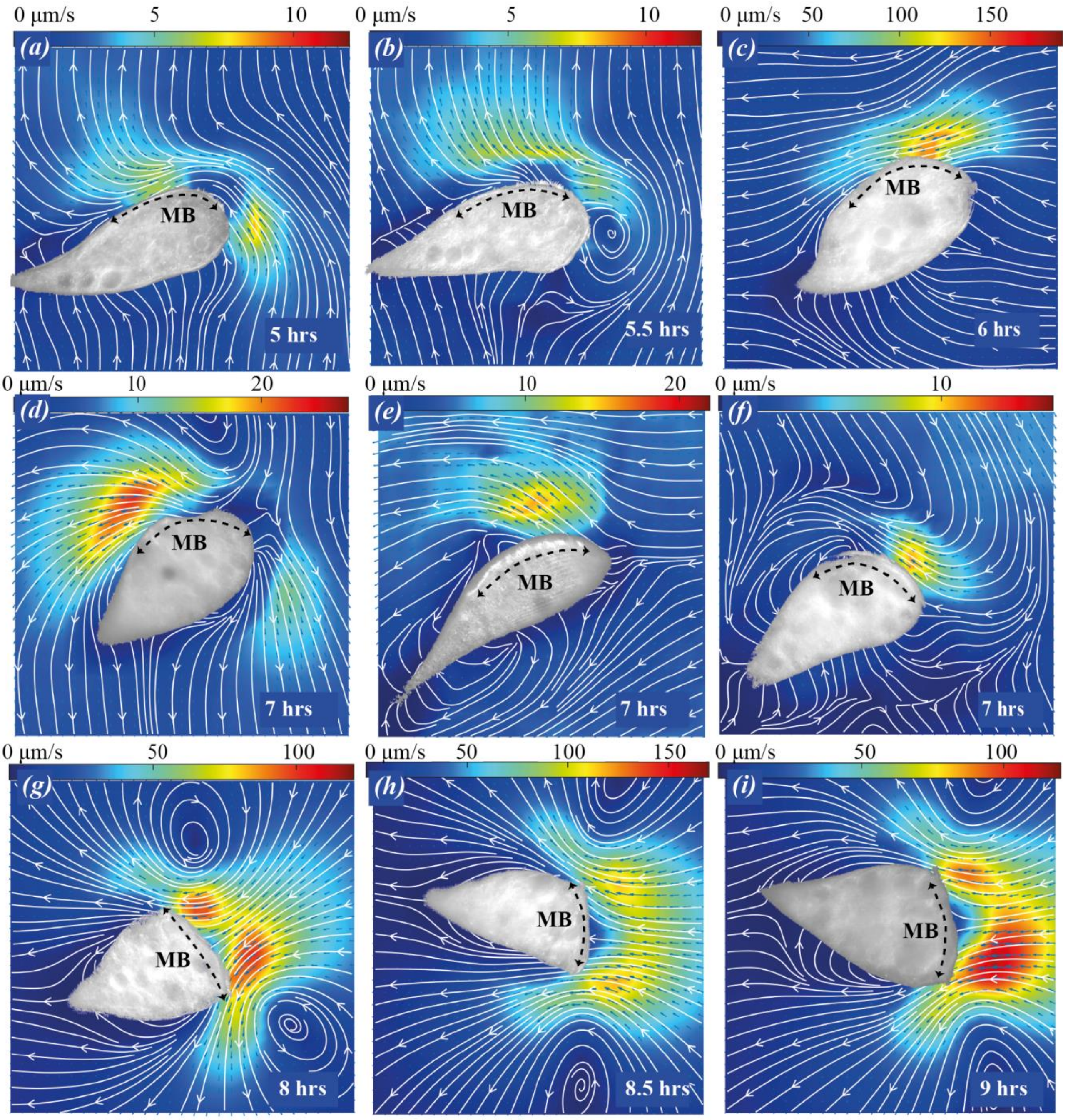

Figure S5: Flow fields measured at different timepoints over the course of regeneration. Time is measured from the moment of MB shedding via sucrose shock.
